# Effect of Aromatherapy Massage With *Foeniculum vulgare* Mill. Seed Essential Oil Compared to Massage on Anxiety, Well‐Being, and Sleep Quality: An Exploratory Randomized Study

**DOI:** 10.1002/hsr2.72436

**Published:** 2026-05-13

**Authors:** Beatriz Pires Félix, Andreia Rodrigues, Maria João Sousa, Olívia R. Pereira

**Affiliations:** ^1^ Instituto Politécnico de Bragança, Campus de Santa Apolónia Bragança Portugal; ^2^ Centro de Investigação de Montanha (CIMO) Bragança Portugal; ^3^ Laboratório Associado para a Sustentabilidade e Tecnologia em Regiões de Montanha (SusTEC) Instituto Politécnico de Bragança, Campus de Santa Apolónia Bragança Portugal; ^4^ Research Centre for Active Living and Wellbeing (LiveWell) Instituto Politécnico de Bragança, Campus de Santa Apolónia Bragança Portugal

**Keywords:** anxiety, aromatherapy massage, fennel, sleep quality, well‐being

## Abstract

**Background and Aims:**

Aromatherapy is a complementary and alternative medicine (CAM) widely used in the management of several mental and physical conditions. The fennel seed essential oil (FEO) has several biological activities and has been reported as potentially beneficial in the management of anxiety and sleep disorders. The present study aimed to evaluate the effects of aromatherapy massage using FEO compared to massage alone on reducing anxiety and improve sleep quality and overall well‐being.

**Methods:**

FEO was extracted by hydrodistillation, and its chemical composition was determined by GC‐MS. In the aromatherapy trial, 40 participants were randomly divided into the “Massage Group” (*n* = 20) and the “Aroma Massage Group” (*n* = 20). Individuals in the “Aroma Massage Group” were treated with 1.6% of FEO diluted in sweet almond oil, while the “Massage Group” only received the massage with sweet almond oil. The intervention consisted of one massage session every 15 days over a 2 months period. The questionnaires on anxiety (State‐Trait Anxiety Inventory), sleep quality (Pittsburgh Sleep Quality), and well‐being (WHO‐Five Well‐Being Index (WHO‐5)) were performed at four different times. Data were analyzed using SPSS software with a significance level of 5%.

**Results:**

The major compound in FEO was *trans*‐Anethole (78.2%). In the aromatherapy trial, scores on the State Anxiety scale of the State‐Trait Anxiety Inventory (STAI), were significantly lower in the **“**Aroma Massage Group” compared to “Massage Group” with mean values of 32.47 ± 7.86 and 41.75 ± 11.74, respectively, in Questionnaire 3 (after the last session), corresponding to a difference of −9.28 (*p* = 0.007). No statistically significant differences were observed over time across the four assessment points, with *p* = 0.536 for the “Massage Group” and *p* = 0.381 for the “Aroma Massage Group.” Additionally, the “Aroma Massage Group” showed a higher proportion of individuals reporting strong well‐being (89%) and good sleep (32%) compared to the “Massage Group” (65% and 25%, respectively) in Questionnaire 4 (1 month after the end of the intervention), however, these differences were not statistically significant when considering mean questionnaire scores.

**Conclusion:**

This exploratory study suggests that aromatherapy with FEO may be associated with beneficialeffects on anxiety reduction and improvements in well‐being and sleep quality; however, further studies are required to confirm this results.

## 1 Introduction

Phytotherapy aims to use products of plant origin for therapeutic purposes to prevent, alleviate, or treat a pathological condition [1]. Aromatherapy, as a branch of phytotherapy, uses essential oils extracted from plants as complementary and alternative medicine (CAM) with the aim of improving the health and well‐being of the individual's body and mind [2]. It consists of applying the essential oil topically, by inhalation, or, less commonly, orally [3]. Essential oils are synthesized in various plant organs, such as flowers, leaves, seeds, fruits, roots, and rhizomes, and stored in highly specialized structures, such as glandular trichomes, secretory and epidermal cells, cavities, and channels [4]. They are increasingly used in clinical settings to treat different symptoms, including pain, nausea, vomiting and insomnia [5]. They also play an important role in mental health, improving mood, reducing stress and anxiety, and improving sleep quality [6, 7]. Fennel essential oil is described in the literature due to its antioxidant, anti‐inflammatory and antimicrobial properties and is also described as a digestive stimulant and useful in menstrual and stomach cramps. It is also used for flatulence and stimulates milk production due to the galactogenic effect [8, 9]. Besides these properties, studies demonstrate its role in reducing anxiety and improving sleep quality [10, 11, 12]. Considering that anxiety and poor sleep quality are increasingly prevalent conditions associated with modern lifestyles, and given the limited availability of clinical evidence, this study aims to evaluate the effects of aromatherapy administered through massage, compared with massage alone, on the reduction of anxiety, the improvement of sleep quality, and promotion of overall well‐being.

## 2 Methods

### 2.1 Plant Material

The aerial parts and bulb of *Foeniculum vulgare* Mill were collected in Bragança in April 2024, and the dried seeds were obtained commercially from the Celeiro online store.

### 2.2 Essential Oil Extraction

The essential oil from the different parts of *Foeniculum vulgare* Mill was extracted by hydrodistillation for 3 h using a Clevenger apparatus, according to the European Pharmacopeia method [13]. The essential oils were stored at −20°C in vials for further analysis.

### 2.3 GC‐MS Analysis

The identification of the fennel essential oil compounds was performed by Gas Chromatography coupled with Mass Spectrophotometry (GC‐MS) using a Perkin Elmer 600 chromatograph equipped with a DB‐1 fused silica column (30 m × 0.25 mm i.d., film thickness 0.25 μm) and connected to a Perkin Elmer Turbomass Clarus 600 T mass spectrometer. The oven temperature was set from 45°C to 175°C at 3°C per minute, rising to 300°C at a rate of 15°C per minute. Subsequently, the temperature was kept isothermal for 10 min. The injector temperature was set at 300°C, the transfer line to 280°C, and the ionization chamber at 220°C. The carrier gas, helium, had a linear velocity of 30 cm/s. The samples were injected with a flow distribution of 1:40. An ionization energy of 70 eV, an ionization current of 60 μA, and a mass range of 40–300 u were used. Volumes of 0.1 μl were injected with a scan time of 1 s. The essential oil compounds were identified by comparing their retention indices (RI) relative to the C_9_‐C_1__7_ indices of the *n*‐alkanes and their mass spectra with commercial standards and with a library developed based on analyses of reference oils.

### 2.4 Study Participants

The study was randomized and conducted in accordance with the Consolidated Standards of Reporting Trials (CONSORT) 2010 statement [14]. The participants in this study were female and male residents of the Bragança district aged over 18 years, and the sample was obtained by convenience. Individuals with plant allergies and pregnant women were excluded from the study. The 40 participants were randomly allocated to two groups: “Massage Group” (*n* = 20) and “Aroma Massage Group” (*n* = 20) using block randomization (10 blocks of 4). The “Massage Group” received the massage with sweet almond oil, while the “Aroma Massage Group” received the massage with 1.6% diluted FEO in sweet almond oil [10, 15, 16].

**Figure 1:**
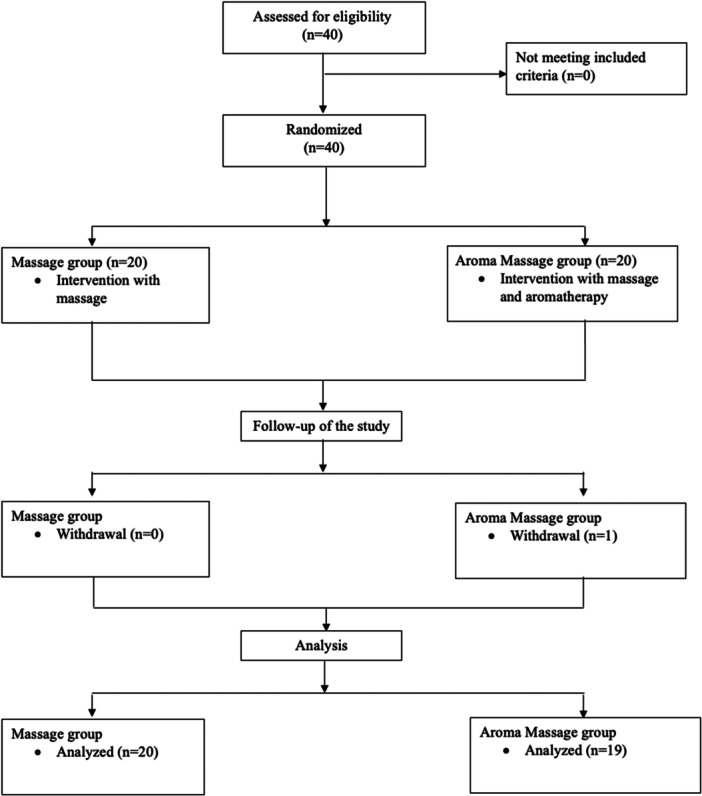
Study flow diagram: recruitment and allocation to study groups.

All bottles were identical in appearance, and the subjects were not informed of the nature of the intervention. Participants were unaware of group allocation; however, complete blinding was not feasible due to the distinctive odor of the FEO. The study flow diagram for recruitment and allocation to study groups is shown in Figure 1.

### 2.5 Measuring Instruments

In the aromatherapy trial, a sociodemographic questionnaire was used to collect participants' age and gender. The Spielberger State‐Trait Anxiety Inventory (STAI) was used to assess anxiety [17]. It consists of two scales with 20 questions each, measuring state anxiety, which refers to the individual's anxiety in response to specific situation, and trait anxiety, which refers to the individual's inherent anxiety. The total score is calculated as the sum of all items and rages from 20 to 80, with responses scored on a 4‐point Likert scale: 1 (not at all), 2 (a little), 3 (moderately) and 4 (very much). To assess well‐being, the WHO‐Five Well‐Being Index (WHO‐5) was used [18], with 5 questions scored from 5 to 0: 5 (all the time), 4 (most of the time), 3 (more than half the time), 2 (less than half the time), 1 (sometimes), and 0 (never). The score calculated by summing the responses, yielding a total ranging from 0 to 25, with 0 corresponding to the worst and 25 to the best well‐being status. Values above 13 indicate a high level of well‐being. The Pittsburgh Sleep Quality Index was used to assess sleep quality [19]. This questionnaire consists of 19 self‐administered questions and 5 partner‐reported questions. The scale is divided into seven components: (1) subjective sleep quality; (2) latency; (3) duration; (4) habitual efficiency; (5) sleep disturbances; (6) use of sleep medication; and (7) daytime dysfunction. Each component is scored between 0 and 3. The global score is calculated by summing the component scores, yielding values ranging from 0 to 21. Scores ≤ 5 indicate good sleep quality, while scores > 5 indicate poor sleep quality.

### 2.6 Intervention and Data Collection

This study was conducted in accordance with the ethical principles for medical research involving human participants (Declaration of Helsinki) and the Oviedo Convention. The aromatherapy trial was registered in Portugal's national registry for clinical studies, RNEC (*Registo Nacional de Estudos Clínicos*), under number 721328. The participants were informed about the study purpose, data confidentiality, and their right to withdraw at any time. Written informed consent was obtained from all participants. The intervention consisted of 30‐minutes sessions conducted every 2 weeks over a period of 2 months, involving a relaxing massage on the back and legs. The “Massage Group” received the massage with sweet almond oil, whereas in the “Aroma Massage Group”, the FEO was administered diluted in sweet almond oil at a concentration of 1.6%. The massage was performed in the same room to ensure consistent environmental conditions across all groups and sessions. Temperature and relative humidity were recorded daily, with medium values of 21.2 ± 1.73°C and 51.8 ± 5.43%, respectively. The questionnaires to assess anxiety, sleep quality, and well‐being were administered at four time points to both groups using the same protocol: before the first session (Questionnaire 1), after the second session (Questionnaire 2), after the last session (Questionnaire 3), and one month after the end of the intervention (Questionnaire 4).

### 2.7 Statistical Analysis

The values from the aromatherapy trial were presented as mean ± standard deviation. The data obtained were analyzed using IBM SPSS Statistics software (version 30). The normality of the results was evaluated using the Shapiro‐Wilk test. Based on the normality assessment, both parametric and non‐parametric methods were applied. When the data satisfied the assumptions of normality, repeated‐measures ANOVA and *t*‐tests were employed; otherwise, the appropriate non‐parametric alternatives, namely the Friedman test and Mann–Whitney *U* test, were applied. A significance level of 5% (*p* < 0.05) was adopted for all analyses.

## 3 Results

The essential oil extracted from the seeds, bulb, and aerial parts had a yield of 1.26%, 0.1%, and 0.3%, respectively. The chemical composition of the different parts of the FEO is shown in Table 1. *trans*‐Anethole was the most abundant compound in the seeds with a percentage of 78.2%, followed by fenchone (9.3%) and methyl chavicol (4.7%), as well as other compounds with lesser percentages, such as limonene and p‐anisaldehyde. The aerial part's most abundant component was methyl chavicol (26.4%), followed by α‐phellandrene (22.5%) and *trans*‐anethole (18.5%). Dillapiole was the most prevalent component in the bulb, accounting for 84.6%, whereas β‐pinene, Y‐terpinene, and α‐phellandrene were present in small quantities (< 3%). In this study, the mean ± standard deviation age of participants in the “Massage Group” was 47.70 ± 15.10 and in the “Aroma Massage Group” it was 53.89 ± 9.12. The demographic and baseline characteristics of the participants are presented in Table 2. No asignificant differences were observed between the two groups (*p* > 0.05) with respect to age, gender, use of sleep medication, state anxiety, trait anxiety, well‐being and sleep quality. Regarding the aromatherapy trial, Table 3 presents the mean (± standard deviation) values from the four questionnaires: Questionnaire 1 (questionnaire administered before the 1st session of the trial), Questionnaire 2 (after the 2nd session of the trial), Questionnaire 3 (after the 4th session of the trial) and Questionnaire 4 (1 month after the last session of the trial) for the “Massage Group” and the “Aroma Massage Group”, as well as mean differences between the two groups. With regard to state anxiety, no statistically significant differences were observed across the different assessment time points within each group, with *p*‐values of *p* = 0.536 in the “Massage Group” and *p* = 0.381 in the “Aroma Massage Group”. In the comparison between the two groups at each assessment time point only in Questionnaire 3 (Q3) was a statistically significant difference observed, with a difference of −9.28 (*p* = 0.007), corresponding to a lower mean anxiety value in the “Aroma Massage Group” than in the “Massage Group”. At the time points, the differences between the groups were not statistically significant, although the “Aroma Massage Group” consistently presented lower mean values; in Questionnaire 1 (Q1), the difference was −3.28 (*p* = 0.342) and in Questionnaire 4 (Q4), it was −3.39 (*p* = 0.270). With regard to trait anxiety, no statistically significant differences were observed in either group across the four assessment time points. Between the two groups, the largest difference observed occurred in Questionnaire 2 (−7.07), although this difference was not statistically significant (*p* = 0.054). Regarding the Well‐Being Index, the analysis of the four questionnaires administered within each group did not reveal statistically significant results, including comparisons between the two groups at each assessment time point. However, the greatest difference was observed in Questionnaire 1, where the “Aroma Massage Group” presented higher scores than the “Massage Group”, with a mean difference of 3.25 (*p* = 0.105). Regarding sleep quality, no statistically significant differences were observed across the questionnaires within each group, nor between the “Massage Group” and the “Aroma Massage Group”, with the largest difference observed in Questionnaire 1, showing a mean difference of −1.39 (*p* = 0.171). The boxplots (see Supplemental Material, Figure 1) ilustrate group trends, score differences, and data distribution. For state anxiety, the median values in the “Massage Group” remain similar from Q1 to Q3, followed by a decrease in Q4. In Q3, the “Aroma Massage Group” showed a lower median value compared to the other questionnaires. The interquartile range was wide in both groups, emphasizing within‐group variability. In trait anxiety, the “Aroma Massage Group” had a lower median score across the four moments compared to the “Massage Group”; however, the interquartile range indicated variability in the results for both groups. In well‐being, the data showed that the “Aroma Massage Group” had higher median values, particularly in Q1, reflecting the group´s high baseline values. In the “Aroma Massage Group,” the median values were similar in Q3 and Q4; however, the median value in the “Massage Group” was lower in Q4 than in Q3. The interquartile ranges were wide in all four questionnaires, particularly in the “Massage Group,” indicating data dispersion. In terms of sleep quality, the “Massage Group” consistently had higher median values over time than the “Aroma Massage Group”. Visually, the “Aroma Massage Group” exhibited smaller value dispersion overall and more stable values in Q4 than the “Massage Group”. Considering the evolution of individuals with strong well‐being (values > 13) (see Supplemental Material, Table 1), it should be noted that in Questionnaire 1, the percentage was 74% (14/19) and 60% (12/20) for the “Aroma Massage Group” and “Massage Group”, respectively, increasing to 89% (17/19) and 80% (16/20) in Questionnaire 3. In turn, in Questionnaire 1, the percentage of individuals with good sleep quality (scores ≤ 5) (see Supplemental Material, Table 1) was 32% (6/19) and 15% (3/20), respectively, increasing in Questionnaire 3% to 55% (10/19 and 11/20) for both groups. In the evolution of individuals with strong well‐being and in the evolution of individuals with good sleep quality, a smaller difference was observed in the two questionnaires in the “Aroma Massage Group”; however, the initial values (Questionnaire 1) were higher in this group. It was also observed that 1 month after the end of the intervention (Questionnaire 4), the percentage of individuals with strong well‐being remained steady in the “Aroma Massage Group” as opposed to the “Massage Group”, where it declined to 65% (13/20). In Questionnaire 4, the percentage of participants with high‐quality sleep decreased less in the “Aroma Massage Group” than in the “Massage Group”, with values of 32% (6/19) and 25% (5/20), respectively.

**Table 1:** Chemical composition of different parts of Foeniculum *vulgare*.

		Seed	Bulb	Aerial part
Compounds	IR			
α‐Thujene	924			0.1
α‐Pinene	930	0.2	0.2	1.4
Camphene	938	t		0.2
Sabinene	958	t	t	0.1
β‐Pinene	963		2.6	1.6
β‐Myrcene	975	0.2	0.1	2.1
α‐Phellandrene	995	t	1.6	22.5
p‐Cymene	1003	0.2	1.6	0.9
β‐Phellandrene	1005	0.1	0.4	2.1
Limonene	1009	3.4	0.7	4.0
*cis*‐β‐Ocimene	1017		t	1.0
*trans*‐β‐Ocimene	1027			0.1
ϒ‐Terpinene	1035	0.1	2.6	0.1
*trans*‐Sabinene hydrate	1037	t		
Fenchone	1050	9.3	0.6	16.0
Terpinolene	1064		0.6	2.0
*cis*‐Limonene oxide	1095	0.3		
Camphor	1102		0.3	0.3
*trans*‐Limonene oxide	1112	0.2		
Terpinene‐4‐ol	1148			0.1
α‐Terpineol	1159			0.1
Methyl chavicol	1163	4.7	0.5	26.4
*trans*‐Carveol	1189	0.1		
α‐fenchyl acetate	1200		0.1	
p‐Anisaldehyde	1210	2.5		
Carvone	1210	t		
*cis*‐Anethole	1220	0.1		0.1
*trans*‐Anethole	1254	78.2	0.3	18.5
Myristicin	1493		0.3	
Kessane	1517		1.4	
Dillapiole	1587		84.6	0.1
Apiole	1640		0.2	
% Identification		99.6	98.7	99.8
RI ‐ Retention index relative to the n‐alkane series C9‐C17				
t ‐ trace (< 0.05%)				

**Table 2:** Demographic and baseline information of participants.

Variables		“Massage Group” (*n* = 20)	“Aroma Massage Group” (*n* = 19)	*p*‐value
Age, mean		47.70 ± 15.10	53.89 ± 9.12	0.129
Sex, *n* (%)	Female	11 (55%)	14 (73.7%)	
	Male	9 (45%)	5 (26.3%)	0.378
Sleep medication, *n* (%)	Yes	7 (35%)	3 (15.8%)	
	No	13 (65%)	16 (84.2%)	0.273
State anxiety		41.65 ± 9.81	38.37 ± 11.45	0.342
Trait anxiety		42.2 ± 9.56	37.11 ± 10.10	0.114
Well‐being Index (WHO‐5)		13.25 ± 3.93	16.5 ± 5.00	0.105
Pittsburgh Sleep Quality Index		8.55 ± 3.94	7.16 ± 3.87	0.171

**Table 3:** Values of state anxiety, trait anxiety, well‐being index (WHO) and sleep quality in the “Massage Group” and “Aroma Massage Group” at different times.

		**“Massage Group”** a **(*n*** = **20)**	**“Aroma Massage Group”** a **(*n*** = **19)**	**Difference in means between groups**	Between‐Group *p*‐value^c^
**State anxiety**	Q1	41.65 ± 9.81	38.37 ± 11.45	−3.28	0.342
	Q2	39.20 ± 11.70	35.53 ± 8.53	−3.67	0.272
	Q3	41.75 ± 11.74	32.47 ± 7.86	−9.28	0.007
	Q4	37.55 ± 10.22	34.16 ± 8.58	−3.39	0.270
	Within‐group *p*‐value^b^	0.536	0.381		
**Trait anxiety**	Q1	42.2 ± 9.56	37.11 ± 10.10	−5.10	0.114
	Q2	41.75 ± 11.91	34.68 ± 10.08	−7.07	0.054
	Q3	40.55 ± 10.80	34.63 ± 9.44	−5.92	0.077
	Q4	38.45 ± 10.81	34.63 ± 9.30	−3.82	0.245
	Within‐group *p*‐value^b^	0.694	0.772		
**Well‐being Index (WHO‐ 5)**	Q1	13.25 ± 3.93	16.5 ± 5.00	3.25	0.105
	Q2	15.55 ± 4.72	15.74 ± 5.10	0.19	0.832
	Q3	15.55 ± 4.98	17 ± 3.61	1.45	0.453
	Q4	14.50 ± 4.90	16.42 ± 3.75	1.92	0.194
	Within‐group *p*‐value^b^	0.116	0.594		
**Pittsburgh Sleep Quality Index**	Q1	8.55 ± 3.94	7.16 ± 3.87	−1.39	0.171
	Q2	7.10 ± 2.97	5.95 ± 3.57	−1.15	0.157
	Q3	5.65 ± 3.12	5.47 ± 3.24	−0.18	0.744
	Q4	7.35 ± 4.32	6.58 ± 4.03	−0.77	0.554
	Within‐group *p*‐value^b^	0.105	0.218		

^a^ Questionnaire values are presented as means ± standard deviation. Q1—Questionnaire 1 (questionnaire before the 1st session of the trial); Q2—Questionnaire 2 (after the 2^nd^ session) Q3—Questionnaire 3 (after the last session); Q4—Questionnaire (one month after the end of the intervention).

^b^ Within‐group *p*‐values represent comparisons across the four time points (Q1–Q4) separately for each group. Repeated Measures ANOVA was used when normality was met; otherwise, the non‐parametric Friedman test was applied.

^c^ Between‐group *p*‐values represent the comparison between the two groups in the four times (Q1–Q4). *T*‐test was used when normality was met; otherwise, the non‐parametric Mann–Whitney test was applied.

No adverse effects were observed during the study.

## 4 Discussion

The chemical composition of the essential oil from the different parts of fennel (seeds, aerial part and bulb) shows several differences, particularly in the major compounds. FEO was used for the aromatherapy trial since the *trans*‐Anethole component is the most abundant in the seeds (78.2%), and several investigations have suggested that this compound may be associated with anxiety reduction [10, 11]. The aromatherapy trial aimed to investigate the effects of FEO through a massage in reducing anxiety and increasing well‐being and sleep quality. It was observed that the values of state anxiety decreased throughout the trial more evidently in the “Aroma Massage Group” in relation to the “Massage Group”, with a statistically significant difference being observed in Questionnaire 3 (after the last massage session) with values of 32.47 ± 7.86 and 41.75 ± 11.74, respectively. This result is in accordance with a study carried out by Alvarado‐Garcia et al. where the use of FEO was associated with a reduction in anxiety levels among university students from 61.45 ± 7.66 to 56.48 ± 5.94 using the Zung Self‐Rating Anxiety Scale [10]. Furthermore, a study carried out on rats by Cioanca et al. demonstrated that the inhalation of FEO at 1% and 3% was associated with anxiolytic‐like effects, with an increase in the percentage of time spent in the open arm and an increase in the number of open arms entries and number of crossings in the elevated plus‐maze test [20]. Although direct comparisons between animal models and human clinical results are not possible due to species differences and the fundamental distinction between behavior testing and self‐reporting, these preclinical findings provide supportive evidence for the potential anxiolytic properties of FEO and help to reinforce the biological plausibility of the trends observed in the present study. Similarly, Perveen et al. reported positive effects of fennel oil in reducing anxiety in rats with an increase in time spent in the open arms, also in the elevated plus‐maze test [11]. Regarding the Well‐Being Index and the Pittsburgh Sleep Quality Index, although there has been an improvement in both study groups, no significant differences were observed in the mean values between the groups, indicating that the “Aroma Massage Group,” which used fennel seed essential oil (FEO), did not demonstrate a significantly greater improvement compared to the “Massage Group”. However, more individuals in the “Aroma Massage Group” were rated as having strong well‐being, 74% in Questionnaire 1% and 89% in Questionnaire 4, and good sleep quality, 32% and 55% in Questionnaires 1 and 3, respectively, suggesting a potential benneficial effect of FEO on these outcomes. A study by Polonini et al. demonstrated an improvement in sleep quality using the Pittsburgh Sleep Quality Index after 3 months of using an intranasal spray with fennel essential oil, although this was combined with other essential oils, which may have contributed to a synergistic effect, and the results could not be directly compared with the findings of this study [12]. Although the effect of massage should not be overlooked, the results of the present study suggest favourable trends in well‐being, sleep quality, and anxiety reduction, supporting the potential of FEO as a complementary therapeutic approach. Considering the growing interest in aromatherapy for managing anxiety and sleep disturbances, further studies are warranted to explore the mechanisms underlying these effects and the potential benefits of essential oils for enhanced efficacy. Essential oils stimulate olfactory chemoreceptors, activating olfactory signaling. This signaling terminates in the upper cerebral cortex, where olfactory sensory neurons transmit electrical impulses to the limbic and hypothalamic regions of the brain via the olfactory bulb and superior olfactory cortex. In this way, a cascade of neurotransmitters and neuromodulators is released, potentially contributing to a feeling of calm and tranquility. With topical application, essential oils are absorbed through the skin, interacting with local tissues or entering the bloodstream where they exert their effects [5]. The primary weakness of this study was the inability to distinguish the specific mechanism of action of an essential oil. Furthermore, participant blinding was compromised due to the strong and distinctive odor of fennel seed essential oil, which may have introduced expectation effects and contributed to the observed results. Another disadvantage was the limited number of aromatherapy sessions and small sample size, which resulted in a low‐power exploratory study. Therefore, in future studies, it would be important to perform the study for a longer period, with more sessions in a larger sample. It will be interesting to carry out this methodology for individuals with other characteristics, such as the elderly or patients with dementia (e.g., Alzheimer's disease), in palliative and/or oncological care.

## 5 Conclusion

The study suggests that aromatherapy with fennel essential oil (FEO) may constitute a non‐invasive, safe, and simple technique with potential anxiolytic effects. Significant differences were identified only for state anxiety. Regarding well‐being and sleep quality, favourable trends were observed but did not reach statistical significance; larger‐scale studies are required for validation. Given the exploratory nature of the study and considering the presence of methodological limitations—including the small number of participants and the absence of a placebo group—further research with larger samples and more rigorous methodological designs is required to validate and extend these findings, elucidate the underlying mechanisms, and clarify the potential role of fennel essential oil in reducing anxiety and promoting sleep quality and overall well‐being.

## Author Contributions

**Beatriz Pires Félix:** investigation, writing – original draft, methodology, data curation. **Andreia Rodrigues:** investigation, methodology, writing – review and editing, resources. **Maria João Sousa:** conceptualization, funding acquisition, methodology, writing – review and editing, resources, supervision. **Olívia R. Pereira:** supervision, formal analysis, methodology, writing – review and editing, conceptualization, funding acquisition, data curation. The authors read and approved the final manuscript.

## Conflicts of Interest

The authors declare no conflicts of interest.

## Transparency Statement

The lead author Olívia R. Pereira affirms that this manuscript is an honest, accurate, and transparent account of the study being reported; that no important aspects of the study have been omitted; and that any discrepancies from the study as planned (and, if relevant, registered) have been explained.

## Supporting information

Supporting File 1

Supporting File 2

## Data Availability

The data that support the findings of this study are available from the corresponding author upon reasonable request.
