# Supplementary material for: Effect of Aromatherapy Massage With Foeniculum vulgare Mill. Seed Essential Oil Compared to Massage on Anxiety, Well‐Being, and Sleep Quality: An Exploratory Randomized Study
Source: Health Sci Rep. 2026 May 13;9(5):e72436. doi: 10.1002/hsr2.72436 (PMC13172767; doi:10.1002/hsr2.72436)
Supplement: Supplementary file 1 — Supporting File 1 [file HSR2-9-e72436-s001.pdf]

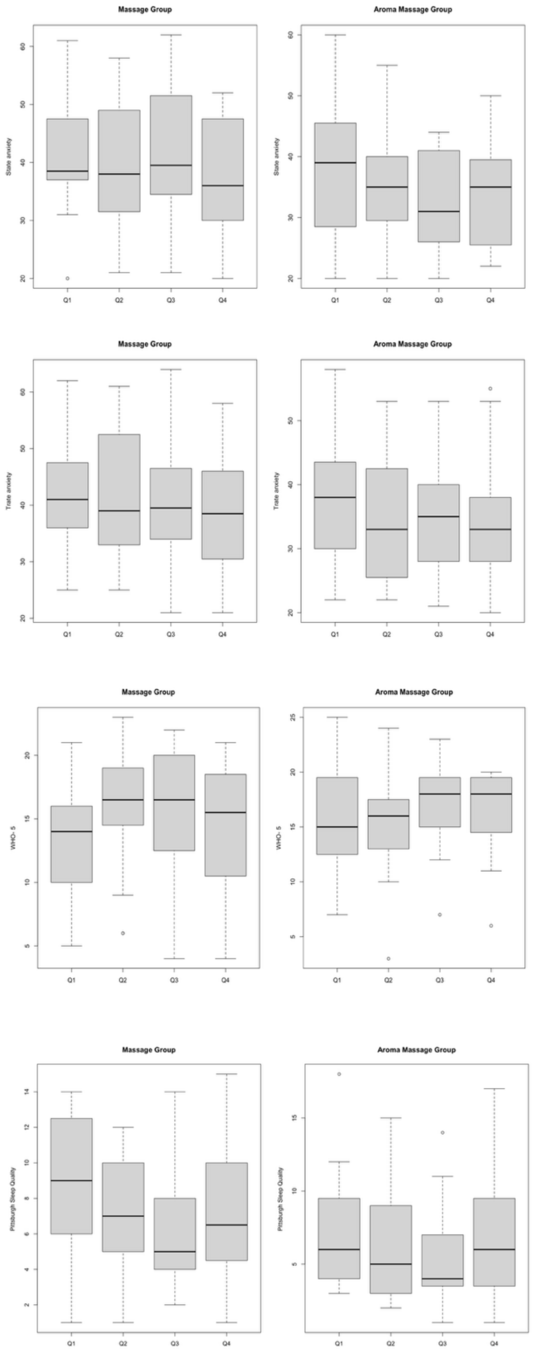

A)

B)

C)

D)

FIGURE 1: Distribution of scores for A) State Anxiety; B) Trait Anxiety; C) Well-being Index (WHO); D) Sleep Quality at four assessment points, Questionnaire 1 (Q1), Questionnaire 2 (Q2), Questionnaire 3 (Q3) and Questionnaire 4 (Q4) for the "Massage Group" and "Aroma Massage Group".
