# Supplementary material for: Effect of Aromatherapy Massage With Foeniculum vulgare Mill. Seed Essential Oil Compared to Massage on Anxiety, Well‐Being, and Sleep Quality: An Exploratory Randomized Study
Source: Health Sci Rep. 2026 May 13;9(5):e72436. doi: 10.1002/hsr2.72436 (PMC13172767; doi:10.1002/hsr2.72436)
Supplement: Supplementary file 2 — Supporting File 2 [file HSR2-9-e72436-s002.pdf]

TABLE 1: Evolution of individuals with strong well-being and good sleep quality in "Massage Group" and "Aroma Massage Group" in Questionnaires 1,2,3 and 4.

	**Questionnaires**	**"Massage Group"**	**"Aroma Massage Group"**
**Evolution of individuals with strong well-being (%)**	**Q1**	60	74
	**Q2**	80	84
	**Q3**	80	89
	**Q4**	65	89
**Evolution of individuals with good sleep quality (%)**	**Q1**	15	32
	**Q2**	15	47
	**Q3**	55	55
	**Q4**	25	32

Q1- Questionnaire 1 (before the first session); Q2- Questionnaire 2 (after the second session)
Q3- Questionnaire 3 (after the last session); Q4- Questionnaire 4 (one month after the end of the treatment).
